# Digital oral health biomarkers for early detection of cognitive decline

**DOI:** 10.1186/s12889-023-16897-w

**Published:** 2023-10-09

**Authors:** Ping-Chen Chung, Ta-Chien Chan

**Affiliations:** 1 Department of Dentistry, Puzi Hospital, Ministry of Health and Welfare, Chiayi, Taiwan; 2 Research Center for Humanities and Social Sciences, Academia Sinica, 128 Academia Road, Section 2, Nankang, Taipei, 115 Taiwan; 3 Institute of Public Health, School of Medicine, National Yang Ming Chiao Tung University, Taipei, Taiwan; 4 Department of Public Health, College of Public Health, China Medical University, Taichung campus, Taichung City, Taiwan; 5 School of Medicine, College of Medicine, National Sun Yat-sen University, Kaohsiung, Taiwan

**Keywords:** Social interaction, Masticatory ability, Periodontitis, Cognitive decline, Oral frailty

## Abstract

**Background:**

Oral health could influence cognitive function by stimulating brain activity and blood flow. The quantified oral status from oral inflammation, frailty and masticatory performance were rarely applied to the cognitive function screening. We aimed to adopt non-invasive digital biomarkers to quantify oral health and employ machine learning algorithms to detect cognitive decline in the community.

**Methods:**

We conducted a prospective case-control study to recruit 196 participants between 50 and 80 years old from Puzi Hospital (Chiayi County, Taiwan) between December 01, 2021, and December 31, 2022, including 163 with normal cognitive function and 33 with cognitive decline. Demographics, daily interactions, electronically stored medical records, masticatory ability, plaque index, oral diadochokinesis (ODK), periodontal status, and digital oral health indicators were collected. Cognitive function was classified, and confirmed mild cognitive impairment diagnoses were used for sensitivity analysis.

**Results:**

The cognitive decline group significantly differed in ODK rate (*P* = 0.003) and acidity from SILL-Ha (*P* = 0.04). Younger age, increased social interactions, fewer cariogenic bacteria, high leukocytes, and high buffering capacity led to lower risk of cognitive decline. Patients with slow ODK, high plaque index, variance of hue (VOH) from bicolor chewing gum, and acidity had increased risk of cognitive decline. The prediction model area under the curve was 0.86 and was 0.99 for the sensitivity analysis.

**Conclusions:**

A digital oral health biomarker approach is feasible for tracing cognitive function. When maintaining oral hygiene and oral health, cognitive status can be assessed simultaneously and early monitoring of cognitive status can prevent disease burden in the future.

## Background

Cognitive decline can result in mild cognitive impairment (MCI) and dementia [1]. Identifying cognitive decline is important for effective intervention and timely prevention of MCI and dementia progression. Early cognitive decline detection aids in avoiding subsequent medical and socioeconomic burdens. Several personal characteristics, including age, sex, education, social connectivity, and comorbidities such as hypertension (HTN), dyslipidemia, diabetes mellitus (DM), depression, anxiety, cardiovascular disease and cerebrovascular disease, oral inflammation, history of cerebral infarction, and cerebral hemorrhage have been identified as important risk factors for MCI [2, 3]. Oral frailty has been associated with an increased risk of new-onset MCI [4]. Previous studies have revealed that the number of teeth present, occlusal contact area, and maximum bite force are significantly higher in cognitively normal subjects than in impaired subjects [5, 6]. Lee et al. pointed out that masticatory function measured by the objective mixing ability test was significantly associated with MCI [6]. In a Japanese 5-year longitudinal study conducted for ≥ 75-year-old residents, after adjusting for the follow-up period, a larger decrease in mini-mental state examination (MMSE) scores was found in elderly individuals with severe periodontitis [7]. Several hypotheses have been proposed to explain the association between oral health and MCI. First, mastication increases neuronal activity and cerebral blood flow [8], and poor masticatory performance, especially tooth loss, is associated with smaller gray matter volume [9]. Secondly, poor masticatory performance also causes dietary changes and malnutrition, which results in cognitive decline [10, 11]. Furthermore, periodontitis is associated with both local and systemic inflammatory responses and increases the risk of Alzheimer’s disease [10]. An increase in oral frailty may cause deterioration in cognitive functions [12].

Many tools have been developed to screen for cognitive disorders, including positron emission tomography, magnetic resonance imaging, MMSE, and the Dementia Rating Scale; these tools have different accessibilities, costs, examination times, and accuracy [13].

Digital health can allow users to regularly monitor and track their health conditions, have quicker access to health services, and potentially prevent diseases and lower healthcare costs. The digital health approach combines technology and healthcare and includes the use of wearable devices, mobile health, telehealth, health information technology, and telemedicine [14].

In this study, we attempted to use digital biomarkers to explore the association between cognitive decline and oral health status through a digital saliva testing instrument, image recognition of a chewing gum test, diadochokinesis testing, and other sociodemographic information. The symptoms of early cognitive decline are not obvious, and early intervention can prevent or slow the progression of dementia. Therefore, we aim to provide a non-invasive screening tool that detects signals of cognitive decline in the community through our digital health approach.

## Methods

### Participants

**Fig. 1:**
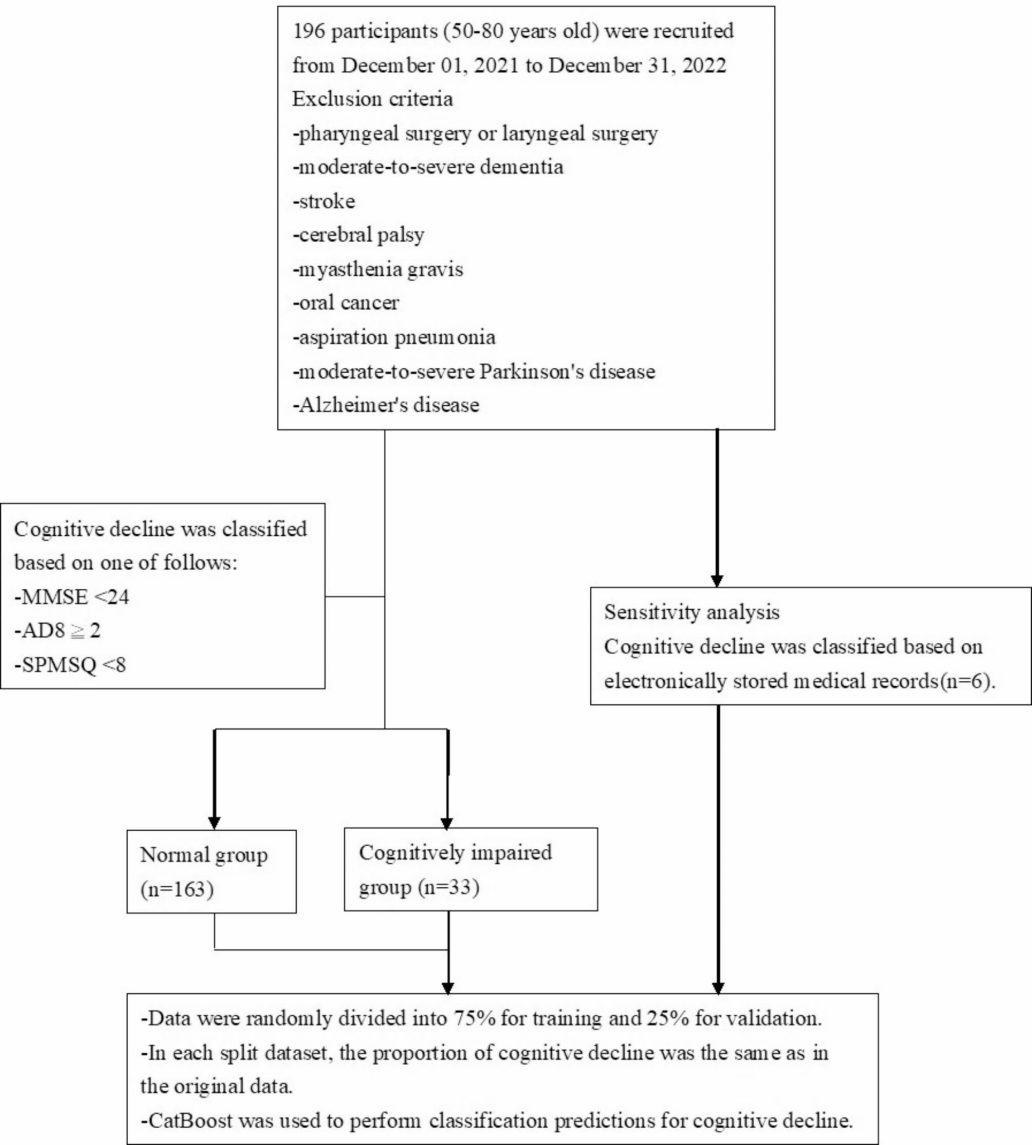
The flowchart of selecting participants

Adult participants between 50 and 80 years old were recruited from Puzi Hospital, (Chiayi County, southern Taiwan) between December 01, 2021, and December 31, 2022. Participants were excluded based on past medical history including pharyngeal surgery or laryngeal surgery, moderate-to-severe dementia, stroke, cerebral palsy, myasthenia gravis, oral cancer, aspiration pneumonia, moderate-to-severe Parkinson’s disease, and Alzheimer’s disease. The flowchart of selecting participants is shown in Fig. 1.

### Data collection

The research team had one clinical dentist and one trained research nurse. Data included demographic characteristics, number of daily interactions, electronically stored medical records, masticatory ability, plaque index, oral diadochokinesis (ODK), stage of periodontitis, and SILL-Ha^®^ saliva test system.

The Eight-item Informant Interview to Differentiate Aging and Dementia (AD8) is an 8-item instrument that differentiates cognitive function by assessing memory, temporal orientation, judgment, and functioning [15]. The Chinese AD8 was validated in Taiwan with a cutoff value of 2 for discriminating between nondemented individuals and those with very mild dementia, yielding an AUC of 0.948, sensitivity of 95.9%, and specificity of 78.1% [16]. The MMSE, a 30-point questionnaire, measures cognitive impairment by evaluating orientation, repetition, verbal recall, attention and calculation, language, and visual construction domains [17]. In the Chinese version of the MMSE, a cutoff value of 24 is used to screen for mild cognitive impairment in individuals with an education level above junior high school degree [18]. The Short Portable Mental State Questionnaire (SPMSQ) is a 10-item list that tests orientation to time and place, memory, current event information, and calculation. A score with 0–2 errors indicates no cognitive decline [19]. In a Taiwan community dementia screening study, the combination of two screening tools, AD8 and MMSE, yielded higher sensitivity and specificity for the early detection of dementia compared to using them separately [20]. We used combination of the above three tools to differentiates cognitive function. Cognitive decline was classified as meeting one of the following criteria: MMSE < 24, AD8 ≥ 2, and/or SPMSQ < 8. The normal group did not meet these criteria (Fig. 1). In a nationwide in-person interview survey conducted between December 2011 and March 2013, 10,432 participants aged 65 years and older were randomly selected from Taiwan. The standardized prevalence of MCI in rural, suburban, and urban areas was 20.29% (95% confidence interval (CI), 20.28–20.29%), 16.67% (95% CI, 16.66–16.67%), and 15.11% (95% CI, 15.11–15.12%), respectively [21]. This reported prevalence is similar to our study’s finding of 16.8% for MCI. The optimal participant ratio for the case-control study was 1:1. However, recruiting large numbers of patients may not be feasible in many situations. If we have a limited number of cases, we can use a ratio of four controls to one case to enhance the statistical power [22]. The study design was followed the STROBE guideline.

### Masticatory ability

**Fig. 2:**
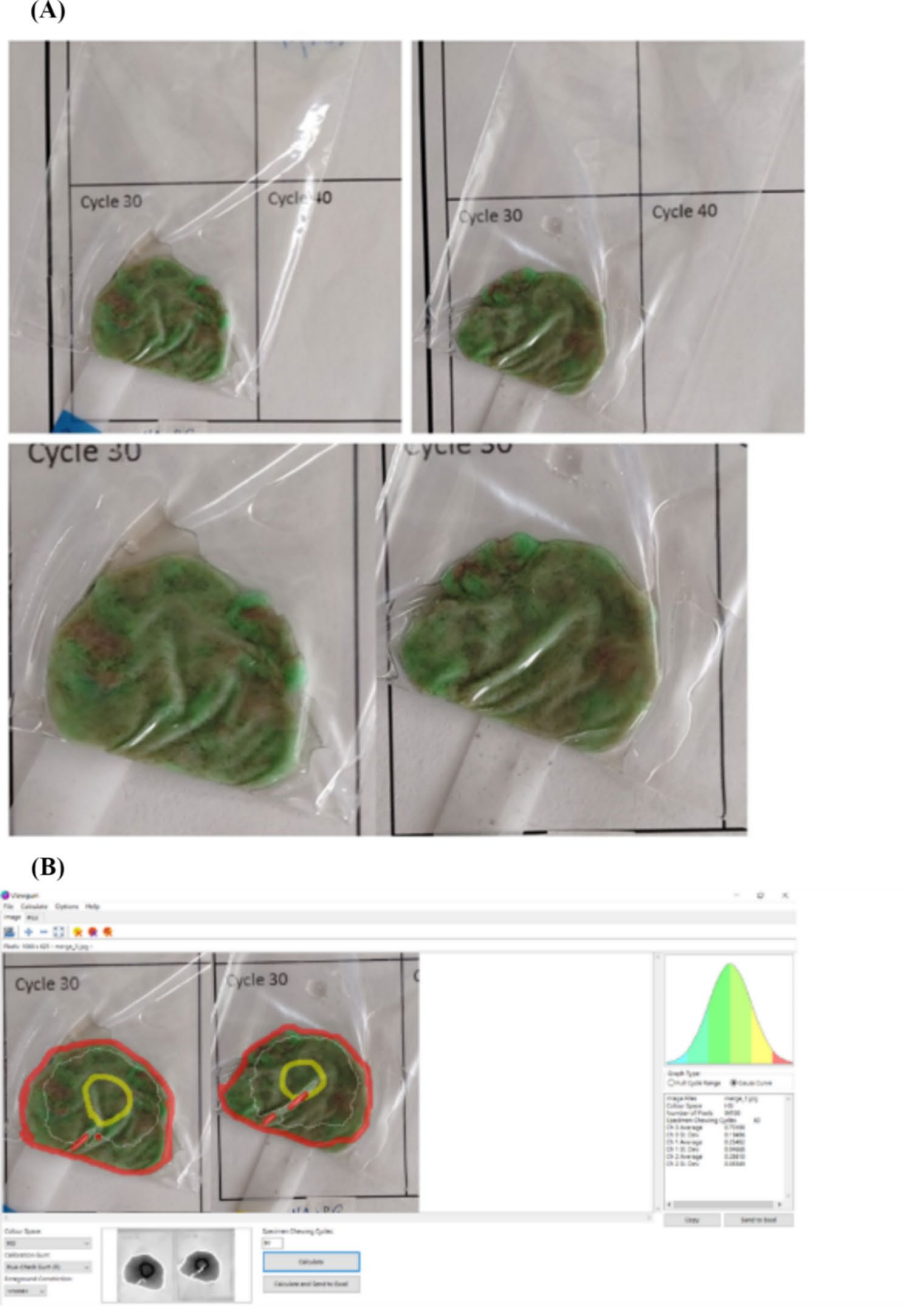
The processing and image identification of chewed gum samples. **(A)** The example of chewed gum samples placed in a transparent plastic bag, and flattened to 1 mm using a plastic plate. **(B)** The images were transformed into hue (H), saturation (S), and intensity (I) by image identification process

A two-colored gum comprising a green and a dark violet layer was produced by Vivident Fruit Swing Karpuz, Turkey. Participants chewed for 30 chewing cycles as counted by the research nurse. The number of cycles based on our pilot study found that 30 were sufficient to show color differences in the gum. The chewed gum samples were retrieved, placed in a transparent plastic bag, and flattened to 1 mm using a plastic plate (Fig. 2A). Both sides of the samples were photographed on-site using a smartphone (HTC Desire 20+). The images were transformed into hue (H), saturation (S), and intensity (I) using the freely available software ViewGum (Fig. 2B, [23]). The variance of hue (VOH) reflects the degree of mixing, with a larger VOH indicating inadequate mixing.

### Plaque index

The plaque index was assessed using the O’Leary plaque control record, obtained by examining six dental surfaces of all teeth and calculating the percentage of dental surfaces with dental plaque [24].

### Oral diadochokinesis

Oral diadochokinesis (ODK) evaluates the function of the lips, anterior tongue, and posterior tongue [25]. ODK was measured by computing the time taken to produce a multi-syllable sound (/pataka/) ten times and was converted to ODK rate by calculating the number of syllables pronounced per second.

### Stage of periodontitis

According to the 2017 World Workshop on the Classification of Periodontal and Peri-Implant Diseases and Conditions [26], periodontitis is classified mainly by interdental clinical attachment loss, radiographic bone loss, and tooth loss assist classification.

### SILL-Ha^®^ saliva test

Each participant was instructed to rinse their mouth with 3 mL of distilled water for ten seconds. The discharged oral rinse solution was dropped onto each of the seven pads of the test strip supplied with the kit. The strip was placed into the device (SILL-Ha^®^ ST-4910; Japan), which analyzed seven indicators for tooth health (acidity, buffering capacity, and cariogenic bacteria), gum health (occult blood, leukocytes, and proteins), and oral cavity cleanliness (ammonia).

### Statistical analysis and data mining

The participants were divided into normal and cognitive decline groups. Age, sex, number of daily interactions, comorbidity including HTN, hyperlipidemia, DM, depression, anxiety, cardiovascular disease and cerebrovascular disease, VOH from bicolor chewing gum, plaque index, ODK rate, stage of periodontitis, and the indicators from SILL-Ha^®^ were examined. Sex is a categorical variable that is treated as a dummy variable and analyzed. Non-parametric Mann–Whitney U test and Fisher’s exact tests were used to compare differences in cognitive status. Data were randomly divided into 75% for training and 25% for validation. In each split dataset, the proportion of cognitive decline was the same as in the original data. In data mining, CatBoost [27] was used to perform classification predictions for cognitive decline. Due to the imbalance in the cognitive status proportion, we adjusted the weight five times for the cognitive decline class in binary classification and prevented overfitting of the machine-learning classifier. In the model-comparison phase, we assessed the predictive performance based on accuracy, precision, recall, f1-score, and area under the curve (AUC) of the receiver operating characteristic curves. The SHapley Additive exPlanations (SHAP) [28], a visualizable approach to explain the output of machine learning model, was used to assess the degree of influence of each explanatory variable on cognitive decline prediction. For sensitivity analysis, we used electronically stored medical records for the most recent year with participants’ informed consent. MCI diagnosis was used for classification, applied to a previously built model, and the model performance was examined. Statistical significance was set at *P* < 0.05. All analyses were performed using R software (version 4.2.2) [29] and Jupyter Notebooks [30].

## Results

In total, 196 participants were studied, including 163 with normal cognitive function and 33 with cognitive decline (Table 1). The majority of participants were female. The average age of the normal group was 68.5 years (standard deviation (SD) = 7.1), and 71.5 years (SD = 6.0) for the cognitive decline group. Faster ODK rates (normal group mean = 4.2, SD = 1.2; cognitive decline group mean = 3.4, SD = 1.5) and lower acidity levels (normal group mean = 64.6, SD = 22.1; cognitive decline group mean = 73.5, SD = 21.0) were significantly more likely in the normal group. Sex, age, number of daily interactions, comorbidity including comorbidity including HTN, hyperlipidemia, DM, depression, anxiety, cardiovascular disease and cerebrovascular disease, mean scores for VOH from bicolor chewing gum, plaque index and stage of periodontitis were not significant difference between normal cognitive function and cognitive decline.

**Table 1:** Demographic and oral characteristics of participants divided by cognitive status

	Normal	Cognitive decline	P-value
	(N = 163)	(N = 33)	
Sex (%)			0.33
Female	97 (59.5)	23 (69.7)	
Male	66 (40.5)	10 (30.3)	
Age, mean(SD)	68.5 (7.1)	71.5 (6.0)	0.03
Interaction, mean(SD)	4.9 (2.9)	4.2 (1.4)	0.64
HTN (%)			
Yes	22 (13.5)	8 (24.2)	0.18
No	141 (86.5)	25 (75.8)	
Hyperlipidemia (%)			
Yes	57 (35.0)	11 (33.3)	1
No	106 (65.0)	22 (66.7)	
DM (%)			
Yes	100 (61.3)	22 (66.7)	0.69
No	63 (38.7)	11 (33.3)	
Depression or Anxiety (%)			
Yes	10 (6.1)	4 (12.1)	0.26
No	153 (93.9)	29 (87.9)	
Cardiovascular disease (%)			
Yes	17 (10.4)	7 (21.2)	0.14
No	146 (89.6)	26 (78.8)	
Cerebrovascular disease (%)			
Yes	7 (4.3)	2 (6.1)	0.65
No	156 (95.7)	31 (93.9)	
Masticatory ability (VOH), mean(SD)	0.2 (0.1)	0.2 (0.2)	0.58
Plaque index, mean(SD)	31.2(19.2)	32.9 (23.6)	0.83
Oral diadochokinesis, mean(SD)	4.2 (1.2)	3.4 (1.5)	0.003
Stage of periodontitis (%)			0.31
Normal	6 (3.7)	0 (0)	
Stage I	53 (32.5)	16 (48.5)	
Stage II	63 (38.7)	9 (27.3)	
Stage III	32 (19.6)	6 (18.2)	
Stage IV	8 (4.9)	1 (3.0)	
Not applicable^a^	1 (0.6)	1 (3.0)	
SILL-Ha^®^, mean(SD)			
Cariogenic bacteria	17.0 (21.1)	20.0 (20.6)	0.26
Acidity	64.6 (22.1)	73.5 (21.0)	0.04
Buffering capacity	10.5 (11.9)	10.7 (16.3)	0.38
Occult blood	35.6 (26.0)	34.2 (27.1)	0.71
Leukocytes	58.5 (31.7)	52.7 (33.3)	0.33
Proteins	32.1 (19.4)	33.1 (22.2)	0.91
Ammonia	21.9 (18.7)	21.6 (23.6)	0.51

^a^ Full mouth edentulous ridges

Abbreviations

SD: standard deviation

HTN: hypertension

DM: diabetes mellitus

VOH: variance of hue

**Fig. 3:**
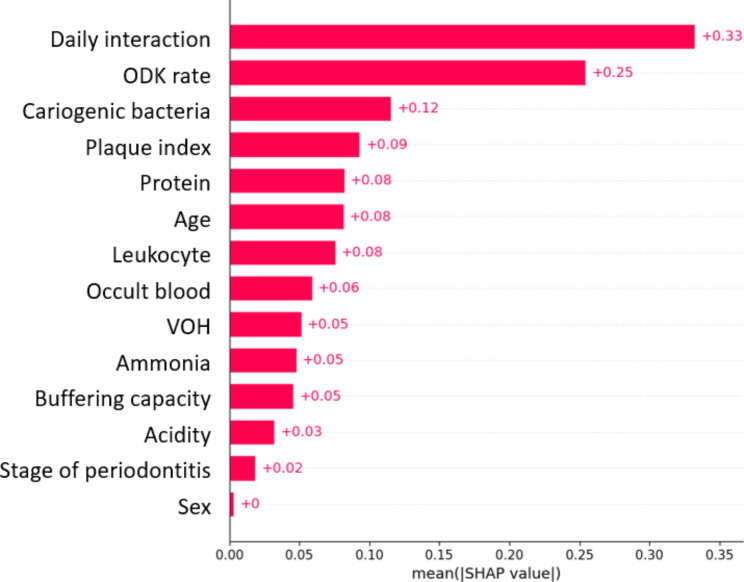
Global feature importance for predicting cognitive decline based on SHAP values. Abbreviations in the figure: SHAP: SHapley Additive exPlanations; ODK: oral diadochokinesis; VOH: variance of hue

**Fig. 4:**
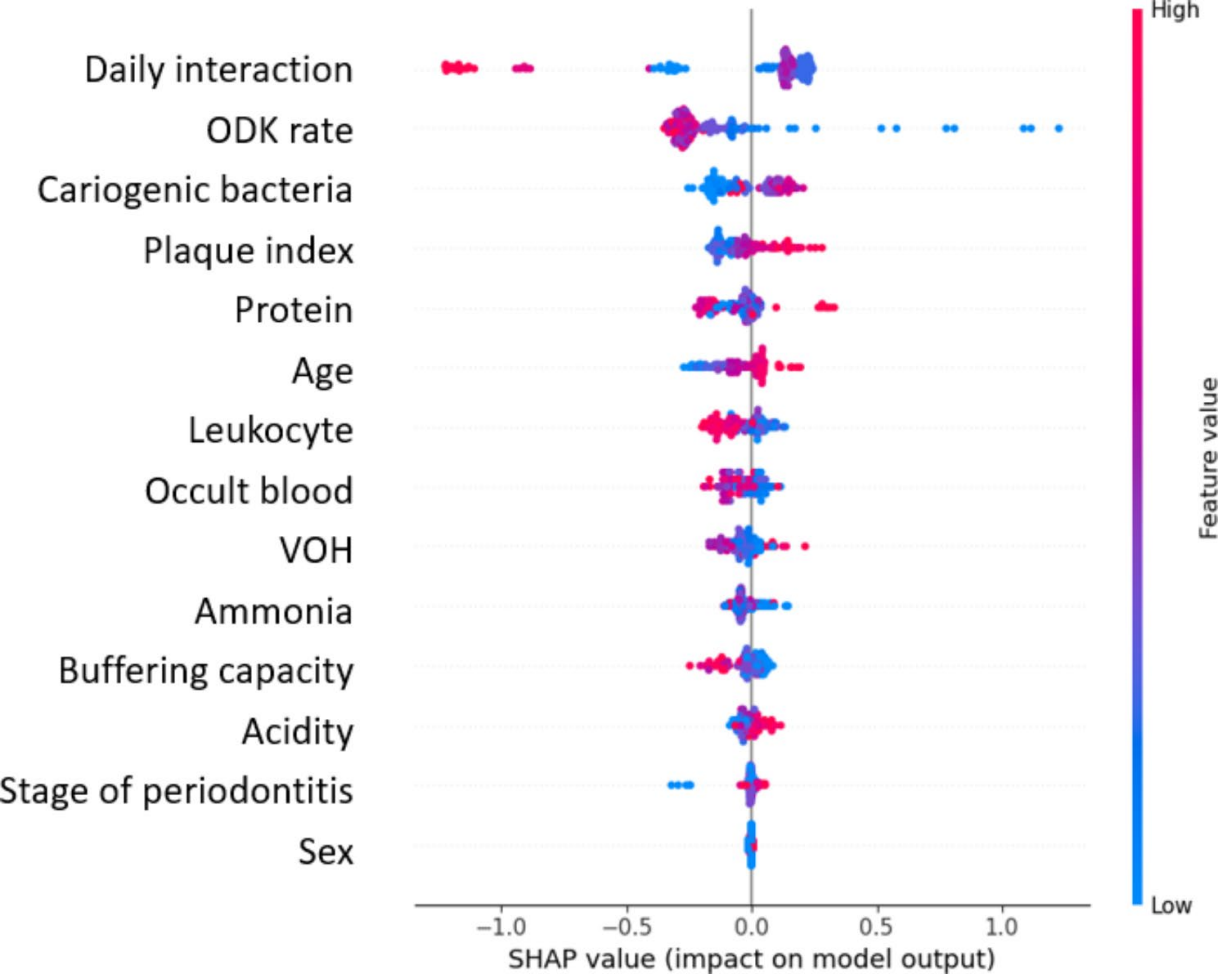
Local explanation summary plot for predicting cognitive decline. Abbreviations in the figure: SHAP: SHapley Additive exPlanations; ODK: oral diadochokinesis; VOH: variance of hue

The SHAP model calculates the marginal contribution of features to the model output and produces a predicted value (SHAP value) to represent the importance of a feature to the prediction model. Red represents higher values of the variable while blue represents lower values of the variable. A positive SHAP value indicates that the corresponding feature contributes to an increased risk of cognitive decline, whereas a negative value indicates that the corresponding feature leads to a lower risk. For the CatBoost classifier, parameters were tuned to achieve better performance, including learning rate = 0.01, maximum depth = 3, number of trees = 300, and the ratio of the number of cognitive decline group to the normal group = 5. The sensitivity, specificity, accuracy-weighted score, precision-weighted score, recall-weighted score, f1-weighted score, and AUC were 0.85, 0.88, 0.86, 0.90, 0.86, 0.87, and 0.86, respectively. Figure 3 shows the ranking of the SHAP value of the CatBoost classifier, which results in the prediction of cognitive decline. The features were ranked according to the sum of the absolute SHAP values for all the samples. Features were ranked in descending order of importance at the global level: Interaction > ODK rate > Cariogenic bacteria > Plaque index > Proteins > Age > Leukocytes > Occult blood > VOH from bicolor chewing gum > Ammonia > Buffering capacity > Acidity > Stage of periodontitis > Sex. An increased number of daily interactions, low cariogenic bacteria, younger age, high leukocytes, and high buffering capacity led to lower risk of cognitive decline. Patients with slow ODK, high plaque index, VOH from bicolor chewing gum, and acidity were associated with increased risk of cognitive decline (Fig. 4). Slow ODK rate, older age, high plaque index and acidity, and being female were associated with increased risk of cognitive decline.

In the sensitivity analysis, there were six participants with clinical confirmed MCI. When this data was applied to the model, the sensitivity, specificity, accuracy-weighted score, precision-weighted score, recall-weighted score, f1-weighted score, and AUC were 0.81, 1.0, 0.82, 0.98, 0.82, 0.88, and 0.99, respectively.

## Discussion

In this study, we adopted innovative digital biomarkers to elucidate the social interactions, chewing ability, and oral health associated with cognitive decline after adjusting comorbidity. Relying on these noninvasive and objective digital biomarkers, high classification accuracy was robust, promising great potential as a screening tool in clinical and community settings.

In a nationwide population-based cross-sectional survey conducted between December 2011 and March 2013, the age-adjusted prevalence of MCI was 18.76% and the prevalence of all-cause dementia was 8.04% among the Taiwanese population aged ≥ 65 years. Age, sex, and education level were significantly associated with MCI and dementia [31]. Cognitive decline was found in 16.8% of our participants, similar to the national MCI prevalence rate.

Social interactions have been shown to protect against dementia and mitigate disease progression. In a longitudinal population-based urban cohort study, among those without cognitive impairment at baseline, those who were socially isolated (fewer than three friends) and lonely had 2.99 (95% CI: 1.00–8.94) odds of incident MCI or dementia than those who were neither socially isolated nor lonely. Frequent phone conversations with friends and family members lowered the odds of developing MCI or dementia [32]. In our study, participants with more daily interactions were less likely to decline in cognitive function. Participants with lower ODK rates were more likely to decline in cognitive function. Elders with decreased oral motor skills, especially lip movement, have less desire to engage in conversation and go out [33].

Digital biomarkers provide an objective and quantifiable approach for long-term follow-up. In a total sample of 34 studies in a systematic review to explore MCI, 14 digital cognitive tests showed a good diagnostic performance using digital cognitive biomarkers for MCI, with a sensitivity and specificity over 0.80 [34]. Digital cognitive testing is an effective tool for the early detection of MCI. Our model also showed good performance with a sensitivity of 0.85 and a specificity of 0.88 for cognitive decline. The sensitivity analysis for participants with confirmed MCI diagnoses also performed well.

Previous studies have shown that masticatory stimulation can stimulate brain activity. Onozuka et al. pointed out that while chewing, blood oxygenation level-dependent signals increase bilaterally in some parts of the brain, including the sensorimotor cortex, supplementary motor area, insula, thalamus, and cerebellum [8]. Another study revealed the influence of chewing on neuronal activity in the brain during a working memory task, using functional magnetic resonance imaging. Gum chewing induces activation of the middle frontal gyrus in the dorsolateral prefrontal cortex and enhances the effect of chewing on cognitive function [35]. In our study, participants with poor masticatory ability and a large VOH were more likely to develop MCI.

Different types of bacteria are present in the oral cavity that have local effects, causing dental caries, plaque deposition, periodontitis, and oral inflammatory diseases. The plaque index correlates well with the number of bacteria, including *Actinomyces viscosus/naeslundii*, *Streptococcus sanguis*, and *Streptococcus mutans* [36]. Dysbiosis of polymicrobial communities induces dysregulated and destructive host responses. Oral bacteria also enter the pulp bloodstream through carious lesions or invade the bloodstream through periodontal pockets and exacerbate systemic inflammation within distant tissues [37]. Acute and chronic systemic inflammation is characterized by the systemic production of proinflammatory cytokine tumor necrosis factor α (TNF-α), mainly from macrophages. TNF-α plays a role in immune-brain communication and increases the rate of cognitive decline, and is associated with reduced hippocampal volume [38]. In our study, participants with a low plaque index or an initial stage of periodontitis were less likely to decline in cognitive function.

Saliva analysis is a noninvasive method that can provide considerable information. The SILL-Ha^®^ test measures the chemical balance of a test strip using dual-wavelength reflectometry; the test strip measures seven saliva factors, including cariogenic bacteria, acidity, buffer capacity, blood, leukocytes, proteins, and ammonia. With repeated measurements using a sample of 20 participants, the intra-class correlation coefficients of the seven saliva factors were 0.67–0.93, above moderate-level reliability [39]. Among 104 adult participants, those with pocket depth measurements > 5 mm had higher levels of leukocytes and proteins than those with pocket depths of ≤ 5 mm [40]. Systemic diseases influenced the results of SILL-Ha^Ⓡ^. For example, participants with diabetes and/or cancer are more likely to have lower leukocyte and higher ammonia levels, and those with sleep apnea are more likely to have lower acidity [40].

In our study, participants with high cariogenic bacteria, low buffering capacity and high acidity were more likely to have cognitive decline. Protein, occult blood levels and ammonia levels were not associated with cognitive decline, however, participants with low leukocyte level were more likely to have cognitive decline. This may be because lymphocyte functions are classified into innate and adaptive immunity. A previous study presented differential roles of innate and adaptive immunity in the incidence of dementia. Increased neutrophil and neutrophil-to-lymphocyte ratios are associated with higher dementia risk, whereas increased lymphocyte and lymphocyte-to-monocyte ratios are associated with lower dementia risk [41].

The present study has some limitations. First, we were uncertain of causality between oral health and cognitive decline because of the cross-sectional nature of this study. Second, despite the similarity in our proportion of MCI to a previous nationwide report, the higher participation rate among individuals with greater health awareness and better health status resulted in a relatively low ratio of cases to controls. Third, we could not classify different types of leukocytes from SILL-Ha^®^. However, this model has good performance on cognitive decline detection. In the future, we could continue to follow up these participants with consent to assess the effectiveness of oral health on cognitive progression and recruit more individuals with clinically confirmed mild cognitive impairment to differentiate between different degrees of cognitive decline and digital oral health indicators measured by our approach.

## Conclusions

To reduce the risk of cognitive decline, it is important to increase social interactions, maintain good oral health to reduce plaque deposition and oral acidity, train oral function, and regularly visit dentists to establish and maintain sufficient occlusal contact. A digital oral health biomarker approach is feasible for tracing cognitive function. When maintaining oral hygiene and oral health, cognitive status can be assessed simultaneously and early monitoring of cognitive status can prevent disease burden in the future.

## Data Availability

The datasets used during the current study are available from the corresponding author on reasonable request.

## List of abbreviations

MCI: Mild cognitive impairment
HTN: Hypertension
DM: Diabetes mellitus
MMSE: Mini-Mental State Examination
ODK: Oral diadochokinesis
AD8: Eight-item Informant Interview to Differentiate Aging and Dementia
SPMSQ: Short Portable Mental State Questionnaire
VOH: Variance of hue
AUC: Area under the curve
SHAP: SHapley Additive exPlanations
SD: Standard deviation
